# Post hoc deconvolution of human mitochondrial DNA mixtures by EMMA 2 using fine-tuned Phylotree nomenclature

**DOI:** 10.1016/j.csbj.2022.06.053

**Published:** 2022-07-02

**Authors:** Arne Dür, Nicole Huber, Alexander Röck, Cordula Berger, Christina Amory, Walther Parson

**Affiliations:** a Institute of Mathematics, University of Innsbruck, Technikerstrasse 13, 6020 Innsbruck, Austria; b Institute of Legal Medicine, Medical University of Innsbruck, Müllerstrasse 44, 6020 Innsbruck, Austria; c MED-EL Elektromedizinische Geräte GmbH, Fürstenweg 77a, Innsbruck 6020 Austria; d synedra IT GmbH, Feldstrasse 1/13, Innsbruck 6020 Austria; e Forensic Science Program, The Pennsylvania State University, 13 Thomas Building, University Park, PA 16802, USA

**Keywords:** Mitochondrial DNA, Mixture Deconvolution, mtDNA Phylogeny, EMPOP, Fluctuation Rates

## Abstract

In this paper we present a new algorithm for splitting (partial) human mitogenomes into components with high similarity to haplogroup motifs of Phylotree. The algorithm reads a (partial) mitogenome coded by the differences to the reference (rCRS) and outputs the estimated haplogroups of the putative components. The algorithm requires no special information on the raw data of the sequencing process and is therefore suited for the post hoc analysis of mixtures of any sequencing technology. The software EMMA 2 implementing the algorithm will be made available via the EMPOP (https://empop.online) database and extends the nine years old software EMMA for haplogrouping single mitogenomes to mixtures with at most three components.

## Highlights

• MtDNA mixtures are observed frequently and difficult to deconvolute.
• Most previous methods require raw data or quantitative information.
• EMMA 2 produces valid splittings from consensus sequences of any sequencing technology.
• EMMA 2 can deconvolute 2 and 3 person mixtures in a fast and traceable way.

## Graphical abstract

**Figure:**
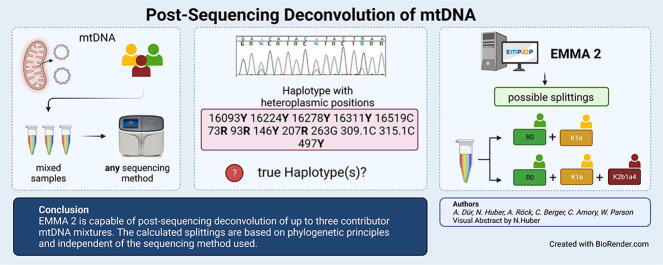

## 1 Introduction

Mitochondrial DNA (mtDNA) enjoys popularity in different fields of research, particularly with the emerging Massively Parallel (Next Generation) Sequencing (MPS) technologies. Full mtDNA genomes (mitogenomes) can meanwhile reliably be generated from minute amounts of ancient DNA [4], challenging samples in the forensic field using a variety of techniques [23], [29], [31] or in medically-oriented studies [34]. In contrast to nuclear DNA that is present in two parentally inherited copies in the nucleus, mtDNA, being located in the mitochondria, is exclusively passed along the maternal lineage and present in many (up to thousands) molecules per cell.

Mixtures of mtDNA are commonly observed in sequencing results and can be assigned to the following sources: first, an mtDNA mixture can consist of contributions from two individuals, either in the form of contamination or as genuine mixture from two individuals, which has particular relevance in forensic genetics [33]. Second, mtDNA is also known to be present in heteroplasmic form within the tissue/cell of an individual [3], [16], [20], which is manifest as mixture of almost identical mtDNA molecules that typically differ only at few nucleotide positions (ntp; mostly between 1 and 3 per individual, [15], [30]. Heteroplasmy is specifically relevant in the medical genetic field at disease–associated positions, where the relative amount of the minor, disease-causing variant can be crucial for the severeness of a disease [34]. A third form is observed as mixture of mtDNA with nuclear mitochondrial elements (NUMTs), whose abundance and frequency have been underestimated with earlier Sanger-based sequencing technologies [11]. The diversity of NUMTs was only recognized when MPS-based technologies were introduced and appropriate identification methods for NUMTs were developed [7]. Finally, one can encounter mtDNA mixtures known to originate from genuine mtDNA and artefacts such as drop-in effects, damage patterns, background signal and more. For more details we direct the reader to a recent review for more in-depth discussion of mixtures from an analytical stand-point [22].

It can be challenging to discern the different forms of mixtures (see [22]), particularly for less experienced analysts. In this study we developed and evaluated a strategy to deconvolute mtDNA mixtures using phylogenetic principles. Earlier attempts to separate human mtDNA mixtures applied physical methods, such as denaturing high-performance liquid chromatography [6], [18], base composition profiling using mass spectrometry [12], quantitative data in a statistical framework [9] and MPS-based data [5], [13], [21] with a continuous statistical phasing framework [28]. While some of these strategies have proven useful, there is still a lack of technology-agnostic, non-quantitative and easy to use mtDNA mixture deconvolution tools that provide traceable splittings on any kind of sequencing data (Sanger and MPS).

## 2 Materials and methods

### 2.1 Mitotypes and symbols

A mitotype or profile describes the nucleotide sequence of an investigated mtDNA fragment as the reading range plus a list of differences to the rCRS [2] coded by the position and the differing symbol. The symbol alphabet has a size of 31 and contains the five unique symbols A,C,G,T and – (the gap for a deletion), and the 26 ambiguous symbols R, Y, S, W, K, M, B, D, H, V, N, a, c, g, t, r, y, s, w, k, m, b, d, h, v, n using the extended IUPAC code [25]. Here, lower-case symbols additionally include the gap. For mitotypes T_1_,T_2_,…,T_k_ the formal mixture T_1_&T_2_&…&T_k_ is the mitotype obtained by joining the symbols of the corresponding sequences at each position. E.g., for T_1_ = 73G 263G and T_2_ = 152C 263G we have T_1_&T_2_ = 73R 152Y 263G. We use the ampersand for mixtures because the plus sign may be contained in the names of the haplogroup motifs, such as H + 195, which denotes the motif of haplogroup H with the additional transition at position 195. Conversely, given an arbitrary mitotype T, we call the representation T = T_1_&T_2_&…&T_k_ with suitable mitotypes T_1_,T_2_,…,T_k_ a splitting of T with components T_1_,T_2_,…,T_k_. For instance, if the query profile has symbol Y at some mtDNA position, the theoretically possible splittings with two components at this position are C&T, C&Y, T&C, T&Y and Y&Y.

### 2.2 Database of haplogroup motifs

The original haplogroup motifs of Phylotree [32] Build 17 from 18 February 2016 were revised in [8]. The revised motifs included 966 additional motifs for yet undetermined subclades. Afterwards we noticed that some additional GenBank mitogenomes of [17] are questionable because of the possible omission of the diagnostic 9 bp-deletion at 8181–8189. Therefore, we deleted all subclades affected by these mitogenomes and provide an updated list of revised motifs in Tables S1-S4. This list of 6380 profiles from 5435 haplogroups consists of 5435 refined haplogroup motifs from Phylotree 17 and 945 additional motifs for subclades. Some motifs carry ambiguous symbols because the corresponding mutations are unstable or uncertain according to Phylotree, e.g., 207R for haplogroup L0f or 16362Y for haplogroup H13b.

### 2.3 The deconvolution problem

For a given mitotype Q and a fixed number k of components the challenge is to find all combinations of mitotypes Q_1_,Q_2_,…,Q_k_ with Q = Q_1_&Q_2_&…&Q_k_ where the components Q_1,_Q_2_,…,Q_k_ resemble haplogroup motifs as close as possible, and the associated haplogroup combinations. Here, differences between the components and the haplogroup motifs may be measured by parsimony or likelihood.

## 3 Calculation

### 3.1 Irredundant extension splittings

To reduce the number of possible splittings without losing relevant splittings, we only consider irredundant extension splittings. Here, the extension condition requires that at every position each unique symbol contained in both the query and the motif symbol is also contained in the symbol of the corresponding component. The irredundancy condition requests that at every position no unique symbol of any component can be omitted without destructing the splitting or violating the extension condition. E.g., for query symbol Y (C and T), component number 2, first motif symbol C and the second motif symbol T, the only irredundant extension splitting is Y = C&T. Contrary, for query symbol B (C, G and T), component number 3, first motif symbol M (A and C), second motif symbol C and third motif symbol G, the three irredundant extension splittings are B = Y&C&G, B = C&Y&G and B = C&C&K, where K is a mixture of G and T.

### 3.2 Log-likelihood ratios

Differences between the components and the haplogroup motifs are quantified by costs, which are sums of log-likelihood ratios (LLRs) of fluctuation rates at each position. For unique symbols fluctuation rates were introduced in [27] as probabilistic rates to measure the fluctuation of positional mutations within haplogroups, and have been refined in [14]. In [27] the LLR of component symbol c and motif symbol m has been defined as log_10_(r(c|c)/r(c|m))/3, where r(c|c) and r(c|m) denote the rates and the normalization of the logarithm has been chosen to obtain LLRs of about 1.0 for standard mutations. E.g., the transition C151T has an LLR of about 0.7, whereas the rarer transversion C16053G has an LLR of about 1.4. In [27] it was shown that for mitotypes minimizing sums of LLRs is an efficient method to maximize the likelihood function.

For an ambiguous symbol in the components the LLR is computed as the arithmetic mean of the LLRs of all unique symbols contained in the component symbol. For an ambiguous symbol in the haplogroup motif the LLR is chosen as the minimal LLR of all unique symbols contained in the motif symbol. E.g., for the component symbol Y and the motif symbol C, we have LLR(Y|C) = LLR(T|C)/2 because LLR(C|C) = 0, while for the component symbol C and the motif symbol Y, we have LLR(C|Y) = 0 for the same reason.

For a perfect splitting, where the components equal haplogroup motifs, the costs are zero. Conversely, if the costs of a splitting are zero, then the components of the query profile differ from haplogroup motifs only by irrelevant mutations. E.g., for the query symbol Y and the motif symbols T and M (A or C) the optimal splitting is Y = T&C with cost LLR(T|T) + LLR(C|M) = 0.

### 3.3 Overview of the deconvolution algorithm

The proposed algorithm is shown in pseudocode in Document S5 and has a single mitotype for the putative mixture and the putative component number as input. Exact genotyping using ambiguous symbols at positions with multiple nucleotides is important because the algorithm relies on the supplied mitotype. Furthermore, the mitotype should be phylogenetically aligned [14] to comply with the haplogroup motifs from Phylotree.

The output is graded by clustering costs of splittings with a margin of 0.5 as for haplogrouping in EMPOP [14]. Splittings have rank 1 if their costs are less than mincost1 + 0.5 where mincost1 denotes the minimal cost among all splittings. Splittings have rank 2 if their costs are equal or greater than mincost1 + 0.5 but less than mincost2 + 0.5 where mincost2 denotes the minimal cost among all splittings that do not reach rank 1. For each rank the algorithm outputs the range of the observed costs, the list of splittings and the list of maximal haplogroup combinations. As the algorithm does not use quantitative information about mixture components, the number of possible splittings can be huge because of the many possibilities of reassigning private mutations of the true components, and displaying long lists of splittings or haplogroup combinations in the output would not be instrumental. Thus, only maximal combinations of haplogroups with respect to the covering relation are listed in Phylotree order [32]. Here, a haplogroup combination is covered by another haplogroup combination if, after a possible reordering, each haplogroup is a subhaplogroup of or equal to the corresponding haplogroup of the other combination. E.g., the two haplogroup combinations R&J and J&U are listed in Phylotree order because R is listed before J and J before U in the tree, but J&U will be omitted in the output, because U is a subhaplogroup of R and thus J&U is covered by R&J. Therefore, when deconvolving mixtures with known components in Section 4, we consider the algorithm successful if the true haplogroup combination is covered by a combination of rank 1 or 2.

### 3.4 Details of the algorithm

The algorithm uses an exhaustive search on the motif combinations to find all optimal or nearly-optimal irredundant extension splittings, has been implemented in C with OpenMP and applies several optimizations described below to achieve acceptable running times for mixtures with at most three components (a mixture of three full mitogenomes takes less than one hour on a custom PC). First, the motif profiles are condensed to the reading range of the query profile to avoid duplicate comparisons. For each condensed database profile its haplogroup is determined as the most common recent ancestor (MRCA) of the haplogroups of the corresponding motif profiles, e.g., for the CR the 6380 haplogroup motifs are condensed to 3439 CR profiles. Second, all possible combinations of motif profiles are tested, which results in about 20 million tests for two components or in about 44 billion tests for three components of full mitogenomes. The algorithm generates all irredundant extension splittings by using precomputed binary tables at every position. These tables have k columns corresponding to the components of the splitting, l rows corresponding to the unique symbols in the query symbol, and entries of 0 or 1. The motif table has the entry 1 if the motif symbol for that component contains the corresponding symbol of the query profile, and 0 otherwise. A table is called an extension table if its entries are 1 whenever the corresponding entries of the motif table are 1. Thus, extension tables are obtained from the motif table by converting zero or more entries from 0 to 1. An extension table is called feasible if each row and each column contains at least one 1, and a feasible extension table is called irredundant if no 1 can be changed to 0 without violating the extension condition or losing feasibility. Then, irredundant extension splittings can be described by irredundant extension tables at every position. In the first example of section 3.1 with query symbol Y (C and T), component number 2, first motif symbol C and the second motif symbol T, the only irredundant extension table is the motif table shown in Table 1a.

**Table 1a:** Motif table for the first example in section 3.1.

Nucleotide	Component 1	Component 2
C	1	0
T	0	1

In the second example of section 3.1 with query symbol B (C, G and T), component number 3, first motif symbol M (A and C), second motif symbol C and third motif symbol G, the three irredundant extension tables can be obtained from the motif table shown in Table 1b by replacing one 0 in the last row by a 1.

**Table 1b:** Motif table for the second example in section 3.1.

Nucleotide	Component 1	Component 2	Component 3
C	1	1	0
G	0	0	1
T	0	0	0

The cost of an irredundant extension splitting is the sum of the LLRs over all positions where query profile or motif profiles differ from the rCRS. The algorithm stops the summation as soon as the current sum exceeds rank2cost + 0.5, because then the splitting cannot reach rank 1 or 2. To decrease the rank 2 costs rapidly the motif profile combinations are generated in the graded lexicographic order of the motifs (A, C, D, …, X, Y, Z, A1, A3, A5, …) such that the combinations of the superhaplogroups are tested first.

## 4 Results and discussion

The interpretation of mixed genetic data deriving from multiple sample donors remains one of the biggest challenges in practical forensic casework. Since the introduction of DNA profiling in the early 1990′s, the forensic community has been tackling this issue in various ways and with varying success. A lot of foreground was produced in mixture deconvolution of nuclear DNA markers for human identification, such as Short Tandem Repeats (STRs) over the years, particularly with the development of concepts and software that support the analyst by providing quantifiable information of possible contributors (see e.g., [10]. In contrast to the Mendelian nature of STR markers, haploid genomes carry linked patterns of signature mutations, especially with the analysis of SNPs, such as mtDNA variants, that can be used to assign mixture components to the contributing sources (e.g., [33], [28]. Here, we apply a new algorithm for the splitting of mtDNA mixtures based on the mitochondrial phylogeny with the major advantage that this concept can be applied to any mtDNA sequences regardless of the technology used. An obvious limitation is the lack of quantitative information on the contributing sources as only the consensus haplotype is used as input for the deconvolution.

Since the concept is based on phylogenetic principles, mixtures are evaluated based on contributing haplogroups rather than haplotypes. The main reason for this is that some mutations, particularly hot-spots, cannot unambiguously be assigned to only one of the possible components. Instead, they can belong to multiple contributors present in a mixture. Hence, the result supplied by EMMA 2 represents a ranked evaluation of possible contributing haplogroups to 2- and 3-person mixtures. As shown in our examples, this strategy provides useful information for the interpretation of a mixed mtDNA profile.

In the following we evaluate the performance of the algorithm EMMA 2 on a variety of artificially created and real-world mixtures, for which the true components are known. We discuss the output in a forensic context that can however also be applied to any other discipline using mtDNA sequences.

### 4.1 Artificial mixtures

To evaluate the algorithm 3751 quality-controlled full mitogenomes from the EMPOP database [24] with different geographic and phylogenetic background were collected, including 1178 Westeurasian, 2036 East Asian, 529 Native American, and 8 Oceanian mitotypes. From this dataset 1000 random mixtures were generated with two components and 100 random mixtures with three components. The deconvolution algorithm was used to compute the maximal haplogroup combinations of ranks 1 and 2 and checked whether the true haplogroup combination was covered by the rank 1 or 2 combinations. We consider the inclusion of the true mitotype combination in the output as successful outcome of this approach as this provides useful information for the analyst and guides further evaluations or experiments (e.g., targeted sequencing of particular positions of interest). Table 2 shows that the true haplogroup combinations were covered in ranks 1 and 2 in almost all mixtures.

**Table 2:** Artificial mixtures deconvolved.

	2 components	3 components
covered by rank 1 combinations	997/1000 (99.7%)	95/100 (95%)
covered by rank 2 combinations	3/3 (100%)	4/5 (80%)

The only mixture, where neither a rank 1 nor a rank 2 combination included the true haplogroup combination, consisted of a Westeurasian haplogroup T2b component with private mutations C151T 309insC G5460A T6488C C14341T, an East Asian haplogroup D4 component with private mutations C150T A8537G A13105G C13983T T16249C, and a Native American haplogroup D1a component with private mutations T152C T195C G5460A T6320C T6378C T13500C C16142T G16213A. The algorithm proposed the optimal splitting with first component from haplogroup T2b + 150 and private mutations C151T 309insC G5460A T6488C C14341T, second component from haplogroup L3c’d and private mutations A8537G C13983T T16249C, and third component from haplogroup D1a and private mutations T195C G5460A T6320C T6378C T13500C C16142T G16213A. This proposed splitting had significantly lower costs than the composition of the true components, because the number of private mutations decreased from 18 to 15 by reassigning private mutations as diagnostic mutations of other haplogroups, i.e., C150T from D4 to T2 + 150, T152C from D1a to L3c'd, and A13105G from D4 to L3c'd.

The results of the artificial mixture experiments aptly demonstrate the general suitability of the EMMA 2 algorithm. The single exception in 1100 tested combinations however pinpoints at a limitation of the approach in those instances, where contributing mitotypes harbor private mutations that constitute signature mutations in other haplogroups. The experimental study shows that this phenomenon may be rare but needs to be kept in mind when interpreting mixtures.

### 4.2 Biological mixtures

In order to evaluate the algorithm with real-world mixtures, we used a variety of different resources that were generated in our laboratory with either Sanger-based or MPS-based technologies (examples 1 and 3) or MPS data that we reviewed for quality control purposes (example 2).

#### 4.2.1 Example Mega-NUMT

The first example is derived from sample IV.3 from [19], where the authors provide evidence for the presence of Mega-NUMTs (multi-copy inserts of full mitogenomes) in the nuclear DNA. The full mitotype listed in the fifth column of Table 2 of [19] has 33 ambiguous symbols spread over the whole mitogenome. Table 3 shows the hypothetical number of components in the first column, the range of costs of rank 1 splittings in the second column, and the haplogroup estimates of EMMA 2 of rank 1 in the last column.

**Table 3:** Haplogroup estimates for IV.3 in rank 1.

Number of components	Costs	Haplogroup combinations
1	20.39–20.86	R
2	2.74–3.19	**V&U4c1**
3	2.74–3.21	R&V&U4c1

In [19] the authors identified this mitotype mixture by cloning and MPS experiments as a mixture of haplogroups V and U4c1, which confirms the EMMA 2 haplogroup estimates in rank 1. The computed two-component splittings of lowest costs 2.74 have components composed of haplogroup V and U4c1 motifs, and five private mutations that can be arbitrarily assigned to the component.

#### 4.2.2 Example short NUMTs

The second example is represented by the full MPS-derived mitotype 64 T 73G 146C 152C 153G 235G 263G 309.1C 315.1C 523del 524del 663G 750G 1438G 1736G 2706G 4248C 4769G 4824G 5237A 7028 T 7049G 8027A 8794 T 8860G 9494R 9506Y 9509Y 9514 W 9522Y 9527Y 9530Y 9540Y 9545R 9548R 9554R 9575R 9578 W 9596C 10958 M 11719A 12007A 12705 T 13638del 13656Y 14766 T 15326G 16111 T 16192 T 16223 T 16227G 16290 T 16319A 16362C that includes 15 ambiguous symbols. In contrast to Mega-NUMTs where ambiguous symbols are expected to be distributed across the entire mitogenome, 13 of the 15 mixed positions occur in the range 9494–9578 here. This suggests the presence of short NUMT sequences overlapping with the genuine mtDNA in this region. Therefore, we applied a modified version of the algorithm with motifs as database profiles only in the first component and NUMTs from [35] as database profiles in the other components. Table 4 shows the hypothetical number of components in the first column, the range of costs of rank 1 splittings in the second column, and the haplogroup/NUMT estimates of EMMA 2 of rank 1 in the last column.

**Table 4:** Haplogroup/NUMT estimates for the second example in rank 1.

Number of components	Costs	Haplogroup combinations
1	23.37–23.85	A2+(64)
2	15.78–16.26	A2+(64)&CDSN1036
3	13.65–14.12	A2+(64)&CDSN660&CDSN1036

Here, CDSN1036 and CDSN660 represent NUMT sequences in the range 9469–9589 with profiles 9477A 9494G 9506 T 9509C 9514 T and 9527 T 9530C 9540C 9545G 9548A, respectively [1]. The splitting proposed by EMMA 2 showed a first component from haplogroup A2+(64) with private mutations T152C 309insC 523-524delAC G5237A A7049G A9596C A10958M 13638delA T13656Y A16227G. The second component presented the NUMT CDSN660 without private mutations, and the third component included the NUMT CDSN1036 with the private mutation A9477G. A review of the bam-file showed that position 9477 (1755 total reads) included 1727 Gs (98.4%), 26 As (1.5%) 1 T (0.1%) and 1 del (0.1%). This example shows that the application of the respective database (human mtDNA motifs or NUMTs) also allows for splitting NUMT mixtures that are commonly observed with MPS analyses [22].

#### 4.2.3 Examples from GEDNAP

As third example two- and three component mixtures generated in the course of the proficiency test program GEDNAP [26] were used to sequence the CR applying Sanger-based sequencing. For each mixture Tables  5a-i (2 persons) and Tables 6a-c (3 persons) show the reported mixed mitotype in the first row, the hypothetical number of components in the first column, the range of costs of rank 1 splittings in the second column, the haplogroup estimates of EMMA 2 of rank 1 in Phylotree order in the last column, and the mitotypes of the single-source components that were known to the laboratory in the last two or three rows. Haplogroup estimates that cover the true haplogroup combinations are indicated in bold-face.

**Table 5:** a-i: Deconvolution and haplogroup estimates for GEDNAP two-component mixtures.

(a): GEDNAP 30 Stain 2 (CR): 16093Y 16172Y 16183 M 16189Y 16209Y 16219R 16278Y 16304Y 16519Y 73R 146Y 185R 263G 291.1a 309.1C 315.1C 456Y 523DEL 524DEL		
1	4.72–5.17	R
2	2.09–2.56	**H5&U6a3a2**, **H5&U6a3 + 185**, **H5&U6a3c**, H5a1j&U6a2a2, H5a1j&U6a5c, H5a1 + 16093&U6a2a2, H5a1 + 16093&U6a5c
3	2.40–2.89	R&H5a1j&U6a3a2, H&H5&U6a3a2, H&H5a1j&U6a3 + 185, H1&H5&U6a, H5&R30a1b&U6a3a2, H5&R30a1b&U6a3 + 185, H5&U6a3a2&U6a3c, H5a1j&H5a1 + 16093&U6a3c, H5a1j&*H*10+(16093)&U6a3c, H5a1j&U6a2a2&U6a3 + 185, H5a1j&U6a3 + 185&U6a3c, H5a1j&U6a3 + 185&U6a5c, H5a1 + 16093&R30a1b&U6a3c, H5a1 + 16093&U6a3 + 185&U6a3c
True component 16209C 16304C 16519C 263G 309.1C 315.1C 456 T 523DEL 524DEL		H5a1j
True component 16093C 16172C 16183C 16189C 16219G 16278 T 73G 146C 185A 263G 291.1A 309.1C 315.1C 523DEL 524DEL		U6a3a2, U6a3 + 185, U6a3c

(b): GEDNAP 30 Stain 4 (CR): 16051R 16086Y 16162R 16214Y 16304Y 16519Y 16527Y 73R 146Y 263G 309.1C 315.1C 456Y		
1	3.98–4.48	R
2	1.99–2.47	**H1a&H5**
3	1.99–2.47	R&H1a3&H5, H1a&H1bt&H5, H1a&H5&U2
True component 16086C 16214T 16304C 146C 263G 315.1C 456T 573.1C 573.2C 573.3C		H5
True component 16051R 16162G 16519C 16527T 73G 263G 309.1C 315.1C		H1a

(c): GEDNAP 32 Stain 3 (16024–573): 16069Y 16092Y 16126C 16153R 16180M 16181M 16182M 16183M 16189Y 16223Y 16266Y 16274R 16362Y 73G 150Y 195Y 228R 263G 295Y 309.1C 309.2C 315.1C 462Y 489C		
1	5.79–6.25	J
2	3.91–4.39	**M&J1c**, G3b1&J1, D4b2b4&J1, **D5&J1**, D6a1&J1
3	3.81–4.31	M&J1c&J2a2, D5a2a+16092&J&J1c, D6a1&J1&J2a2, D6a1&J1c&J2a
True component 16069T 16126C 73G 195C 228A 263G 295T 309.1C 315.1C 462T 489C		J1c
True component 16092C 16153A 16164G 16182C 16183C 16189C 16223T 16266T 16274A 16362C 73G 150T 263G 309.1C 309.2C 315.1C 489C 523DEL 524DEL		D5a2a1+@16172

(d) :GEDNAP 33 Stain 3 (CR): 16183M 16189Y 16209Y 16223Y 16255R 16278Y 16304Y 16519Y 73R 153R 195Y 200R 225R 227R 263G 309.1C 315.1C 456Y		
1	5.27–5.51	L3
2	0.80–1.29	X2c&H5a1j, **X2c1c&H5**, **X2c1c1&H5′36**
3	1.20–1.70	L3′4&X2c1c&H5a1j, L3′4&X2c1c1&H5, L3&X2c&H5a1j, L3&X2c1c&H5, L3&X2c1c1&H5′36, L3f&X2c&H5, L3f&X2c1c&H5′36, X2c&H5&R30a1b, X2c1c&H5′36&R9, X2c1c&H5′36&R30a1b, X2c1c1&R0&R9
True component 16304C 263G 309.1C 315.1C 456T		H5
True component 16183C 16189C 16209C 16223T 16255A 16278T 16519C 73G 153G 195C 200G 225A 227G 263G 309.1C 315.1C		X2c1c1

(e): GEDNAP 33 Stain 4 (CR): 16093Y 16167Y 16171R 16192Y 16223Y 16224Y 16298Y 16311Y 16327Y 16344Y 16357Y 16519C 47R 73G 249del 263G 315.1C 489Y 497Y		
1	5.40–5.86	CZ, M31a1
2	2.16–2.66	**C4a2a&K1a**, C4a2c&K1a
3	1.59–2.09	C4a1b&C4a2a1&K1a, C4a2a&C4a2c&K1a
True component 16167T 16171G 16223T 16298C 16327T 16344T 16357C 16519C 47A 73G 249DEL 263G 309.1C 309.2C 315.1C 489C		C4a2a1
True component 16093C 16192T 16224C 16311C 16519C 73G 263G 315.1C 497T		K1a

(f): GEDNAP 34 Stain 4 (CR): 16092Y 16129A 16147M 16154Y 16172Y 16223Y 16248Y 16320Y 16355Y 16390R 16519C 73R 143R 152Y 182Y 199Y 204Y 234R 263G 309.1C 315.1C		
1	6.58–7.06	N1a1a1a3, N7, N9b1b, H, R30a1
2	1.99–2.43	**N1a1a1a3&H**
3	2.39–2.83	N1a1a1a3&N1a1a1a3&H, N1a1a1a3&N7&H, N1a1a1a3&N9b1b&H, N1a1a1a3&H&H
True component 16129A 16519C 263G 309.1C 315.1C		H+16129, H1cj, H1e+16129, H1j1, H3af, H3b+16129, H63a
True component 16092C 16129A 16147A 16154C 16172C 16223T 16248T 16320T 16355T 16390A 16519C 73G 143A 152C 182T 199C 204C 234G 263G 309.1C 315.1C 573.1C 573.2C 573.3C 573.4C		N1a1a1a3

(g): GEDNAP 35 Stain 4 (16024–573): 16093C 16224C 16311C 16362Y 16519C 73G 263G 315.1C 497T 524.1A 524.2C		
1	0.67–1.16	K1a
2	0.80–1.29	**K1a&K1a**
3	1.20–1.69	K1a&K1a&K1a
True component 16093C 16224C 16311C 16362C 16519C 73G 263G 315.1C 497T 524.1A 524.2C 524.3A 524.4C		K1a5a
True component 16093C 16224C 16311C 16519C 73G 263G 315.1C 497T 524.1A 524.2C		K1a

(h): **GEDNAP 36 Stain 4** (CR): 16093Y 16224Y 16256Y 16311Y 16352Y 16519Y 73R 152Y 263G 309.1C 315.1C 497Y		
1	3.12–3.61	R
2	0.80–1.29	**H&K1a**
3	1.20–1.70	R&R0&K1a
True component 16093C 16224C 16311C 16519C 73G 263G 315.1C 497T		K1a
True component 16256T 16352C 152C 263G 309.1C 315.1C		H14a

(i): **GEDNAP 37 Stain 2** (CR): 16271Y 16298Y 16519Y 72Y 152Y 263G 309.1C 309.2C 315.1C		
1	1.66–1.85	R0
2	1.22–1.70	**R0&HV0**
3	1.62–2.12	R0&R0&HV0
True component 16271C 16298C 72C 263G 309.1C 309.2C 315.1C		HV0
True component 16519C 152C 263G 315.1C		R0

**Table 6:** a-c: Deconvolution and haplogroup estimates for GEDNAP three-component mixtures.

(a): GEDNAP 32 Stain 2 (CR): 16256Y 16270Y 16311Y 16352Y 16399R 73R 152Y 263G 309.1C 315.1C		
1	2.66–2.88	R
2	1.26–1.75	R0&U5a1+@16192, R1&U5a1a1 + 152
3	1.20–1.69	**R&HV&U5a1+@16192**, R0&R1&U5a1a1 + 152
True component 16311C 152C 263G 309.1C 315.1C		H + 152, H1 + 152, H1 + 16311, H13a1 + 152, H13a1a1a, H13a1a2 + 16311, H13a2b1, H14b, H16 + 152, H1bc, H1e1a4, H2 + 152 + 16311, H2a2a2, H3 + 152, H3 + 16311, H30b1, H3q1, H47a, H72, H76, H80, HV + 16311
True component 16256 T 16270 T 16399G 73G 263G 309.1C 315.1C		U5a1+@16192
True component 16256 T 16352C 263G 309.1C 309.2C 315.1C		H14a

(b): GEDNAP 34 Stain 3 (16024–573): 16093Y 16224Y 16278Y 16311Y 16519C 73R 93R 146Y 207R 263G 309.1C 315.1C 497Y		
1	3.38–3.88	R
2	0.80–1.17	H1r1&K1a4h1, H1ao&K1a
3	1.20–1.70	R&HV7&K1a4h1, R&H1r1&K1a4h1, R&H1ao&K1a, R&K1a1b1&K2b1a4, **R0&K1a&K2b1a4, HV7&H&K1a, H&H&K1a, H&K1a&K1a1b1, H&K1a&K1a4h1, H&K1a1b1&K2,** H&K1a4h1&K2
True component 16519C 263G 309.1C 315.1C		R0
True component 16093C 16224C 16311C 16519C 73G 263G 315.1C 497T 524.1A 524.2C		K1a
True component 16224C 16278T 16311C 16519C 73G 93G 146C 207A 263G 309.1C 315.1C		K2b1a4

(c): GEDNAP 37 Stain 3 (CR): 16126Y 16216R 16234Y 16256Y 16270Y 16294Y 16296Y 16298Y 16311Y 16399R 16519Y 72Y 73R 263G 309.1C 315.1C		
1	4.76–5.24	R
2	2.46–2.94	HV0a1&U5a1+@16192, V&U5a1+@16192, V3c&T, V3c&U5a1f1
3	1.80–2.28	**V&T2&U5a1+@16192**, V3c&HV15&U5a1+@16192, **V3c&T&U5a1+@16192**, V3c&T2&U5a1f1
True component 16126C 16294T 16296T 16311C 16519C 73G 263G 315.1C		T2
True component 16234T 16256T 16270T 16399G 73G 263G 309.1C 315.1C		U5a1+@16192
True component 16216G 16298C 72C 263G 309.1C 315.1C		V3c

In GEDNAP 30 Stain 2 (Table 5a) the formal mixture of the true components coincides with the mitotype reported by the lab. The two-component splittings of lowest cost 2.09 have components from haplogroups H5a1j and U6a3a2. As the algorithm also finds splittings into components from haplogroups H5 and U6a3a2 with cost 2.21, the true combination H5a1j&U6a3a2 is not listed in the table, because it is covered by the rank 1 combination H5&U6a3a2.

In GEDNAP 30 Stain 4 (Table 5b) the formal mixture of the true components differs from the mitotype reported by the lab by the variants 309.1c, 573.1c, 573.2c and 573.3c for technical reasons, as it was difficult in this example to interpret C-stretch variation in the mixed haplotype. The two-component splittings of lowest cost 1.99 have haplogroup combination H1a3&H5. The true splitting with haplogroup combination H1a&H5 has cost 2.35 and reaches rank 1. The low cost 1.99 of a hypothetical mixture of three components can be explained by the fact that the motifs of the haplogroups H and U2 differ in the CR only by the transitions 16051G and 73G, and that the true second component harbors both transitions.

In GEDNAP 32 Stain 3 (Table 5c) the formal mixture of the true components differs from the mitotype reported by the lab by the variants 309.2c, 523a, 524c, 16126Y, 16164R and the missing reads 16180 M and 16181 M. This was an extreme mixture as far as the quantitative contribution of the sources were concerned, which is why some of the mixed positions could not be observed in the Sanger sequencing data. The two-component splittings of lowest cost 3.91 have haplogroup combinations D5a2a + 16092&J1c, D5a2a + 16092&J1c5e and D5a2a + 16092&J1c5f. The true splitting with haplogroup combination D5a2a1+@16172&J1c has cost 4.44 and reaches only rank 2 because the transition A16164G, which is diagnostic for haplogroup D5a2a1, has not been detected as a mixture by the lab. The low cost 3.81 of a hypothetical mixture of three components can be explained by the facts that in the splitting with haplogroup combination D6a1&J1c&J2a2, the private mutation G16274A of the second true component becomes a diagnostic mutation of haplogroup D6a1 and that the private mutation T195C of the first true component becomes a diagnostic mutation of haplogroup J2a2.

In GEDNAP 33 Stain 3 (Table 5d) the formal mixture of the true components coincides exactly with the mitotype reported by the lab. The two-component splittings of lowest cost 0.80 have haplogroup combinations X2c1c&H5a1j or X2c1c1&H5, which is the true combination.

In GEDNAP 33 Stain 4 (Table 5e) the formal mixture of the true components differs from the mitotype reported by the lab by the variants 249a, 309.1c and 309.2c for technical reasons. The two-component splittings of lowest cost 2.16 have haplogroup combinations C4a2a1&K1a1a2a, C4a2a1&K1a1 or C4a2a1&K1a. The low cost of a hypothetical mixture of three components can be explained by the fact that the motifs of the haplogroups C4a2a and C4a2c differ in the CR only by the transitions 16344 T or 47A respectively, and that the true first component shows both transitions.

In GEDNAP 34 Stain 4 (Table 5f) the formal mixture of the true components differs from the mitotype reported by the lab by the variants 573.1c, 573.2c, 573.3c and 573.4c for technical reasons. The two-component splittings of lowest cost 1.99 have haplogroup combination N1a1a1a3&H.

In GEDNAP 35 Stain 4 (Table 5g) the formal mixture of the true components differs from the mitotype reported by the lab by the variants 524.3a and 524.4c for technical reasons. The two-component splittings of lowest cost 0.8 have haplogroup combinations K1a1&K1a5a, K1a&K1a5a or K1a1a2a&K1a5a. The low cost of a hypothetical mixture with only one component can be explained by the fact that the motifs of the haplogroups K1a and K1a5a differ in the CR only by the transition 16362C, which can be heteroplasmic, and that both true components exhibit the insertion 524insAC.

In GEDNAP 36 Stain 4 (Table 5h) the formal mixture of the true components differs from the mitotype reported by the lab by the variant 309.1c for technical reasons. The two-component splittings of lowest cost 0.8 have haplogroup combinations H14a&K1a or H14a&K1a1b2a. As the algorithm also finds splittings with haplogroup combination H&K1a of cost 1.26, the true combination H14a&K1a is not listed in the table.

In GEDNAP 37 Stain 2 (Table 5i) the formal mixture of the true components differs from the mitotype reported by the lab by the variants 309.1c and 309.2c for technical reasons. The two-component splittings of lowest cost 1.22 have haplogroup combinations H&HV0 or H&V2c. The true splitting with haplogroup combination R0&HV0 has cost 1.41 and reaches rank 1.

In GEDNAP 32 Stain 2 (Table 6a) the formal mixture of the true components differs from the mitotype reported by the lab by the variant 309.2c for technical reasons. The three-component splittings of lowest cost 1.20 have haplogroup combinations HV&H14a&U5a1a1 + 152, HV&H14a&U5a1+@16192, HV&H14a&U5a1a1 + 152, H14a&R1&U5a1a1 + 152, H3h2&H14a&U5a1a1 + 152, or H14a&H72&U5a1a1 + 152.

In GEDNAP 34 Stain 3 (Table 6b) the formal mixture of the true components differs from the mitotype reported by the lab by the variants 309.1c, 524.1a and 524.1c for technical reasons. The low cost of a hypothetical mixture of two components can be explained by an artificial nearly-perfect splitting of the reported mitotype into two components: the first proposed component 16278 T 16519C 93G 146C 263G 309.1C 315.1C is represented by the motif of haplogroup H1ao together with the private insertion 309insC, and the second proposed component 16093Y 16224C 16311C 16519C 73G 207A 263G 309.1C 315.1C 497 T is represented by the motif of haplogroup K1a4h1 together with the subclade transition 207A and the private insertion 309insC.

In GEDNAP 37 Stain 3 (Table 6c) the formal mixture of the true components differs from the mitotype reported by the lab by the variant 309.1c for technical reasons. The three-component splittings of lowest cost 1.80 have haplogroup combinations V3c&T2&U5a1+@16192 or V3c&T2g1&U5a1+@16192.

The results of the GEDNAP samples demonstrate the strength but also pinpoint limitations of the algorithm. GEDNAP samples are generally considered authentic forensic samples, as they derive from a proficiency testing program that provides specimens that closely mimic real-world casework samples. There, unbalanced mixtures occur that do not show the mixture at all theoretically affected positions. This is particularly true for Sanger sequencing that is not a fully quantitative method. Hence, missing variants obviously affect haplogrouping and thus may have an impact on the ranked presentation of haplogroup combinations. Also, homopolymeric stretches that may be already challenging to interpret in single source data result in complex constellations in mixtures that cannot be unambiguously called and thus also may affect haplogrouping. Still, the true components of the GEDNAP mixtures were included in rank 1 and 2 assignments by the algorithm for all examples.

We further observed that the true haplogroup combination was not always explicitly stated in the output, but in some cases masked by the coarser haplogroup combination. This is due to the applied irredundancy setting that minimizes the reported combinations in a convenient way. Also, we note that 3-person mixtures may yield lower costs than true 2-person mixtures, when shared mutations can be assigned in a different, “cheaper” way. This is also true when private mutations are recognized as signature mutations in other haplogroups as observed above. Still, in the vast majority of cases, the splitting suggested by EMMA 2 is including the true haplogroups in the suggested combinations, which constitutes a useful outcome for further forensic interpretation.

## 5 Conclusions

Mixtures of mtDNA occur frequently, either from two contributing individuals, from NUMTs, as heteroplasmic events, as modifications of mtDNA during aging or as artefacts as a result of the analytical process. Some of these mixtures display similar or even identical phenotypes in the raw sequencing data and can therefore not easily be differentiated by the analyst. While heteroplasmy generally leads to less affected positions in the raw data and is restricted to known hot-spot positions in the CR, Mega-NUMTs and genuine mtDNA mixtures generally affect multiple positions at haplogroup-diagnostic sites. These measures alone are however not sufficient to discern the contributing sources of mixtures easily by hand.

We here present a new algorithm that will be available in EMMA 2, a software implemented in the EMPOP database [24] that produces splittings based on phylogenetic principles. There are several advantages to this concept: the algorithm can be used with any kind of sequencing data, regardless of the sequencing chemistry and technology used. It does not require quantitative data, which are sometimes difficult to extract from the raw data, e.g., from BigDye Terminator Sanger-based technologies. The algorithm was optimized with respect to computing time and resources and can be performed in reasonable time on a custom PC. Furthermore, the algorithm is not hidden in a “black box”, but leads to traceable results based on the concept outlined here. This lends this algorithm a suitable post hoc tool for the analysis of general mtDNA sequencing data.

The splitting results, albeit confirmatory of the input mitotypes in most cases shown here, cannot be taken at face value without considering alternative options. Our evaluation demonstrates that (an excess of) private mutations can lead to alternative splittings that result in lower costs and will thus be favored by the algorithm over the true mixture components. This can however be recognized by the analyst, as haplogroup diagnostic and private mutations are provided in the output.

Our results show that the algorithm is generally reliable. It thus adds to existing suite of mixture deconvolution tools that use other concepts and sources, e.g., quantitative data, to identify the components. More research is needed in terms of documenting and cataloguing mtDNA variation to further improve phylogeny-based algorithms. The proposed algorithm is also applicable to NUMTS or to non-human mtDNA if a representative database such as the 6380 revised haplogroup motifs of Phylotree 17 for human mtDNA is available.

## CRediT authorship contribution statement

**Arne Dür:** Conceptualization, Methodology, Validation, Software, Writing – original draft. **Nicole Huber:** Validation, Writing – review & editing, Software, Data curation. **Alexander Röck:** Validation, Writing – review & editing, Software, Data curation. **Cordula Berger:** Writing – review & editing. **Christina Amory:** Writing – review & editing. **Walther Parson:** Conceptualization, Writing – review & editing, Supervision.

## Declaration of Competing Interest

The authors declare that they have no known competing financial interests or personal relationships that could have appeared to influence the work reported in this paper.
