# Supplementary material for: Post hoc deconvolution of human mitochondrial DNA mixtures by EMMA 2 using fine-tuned Phylotree nomenclature
Source: Comput Struct Biotechnol J. 2022 Jul 2;20:3630–8. doi: 10.1016/j.csbj.2022.06.053 (PMC9283771; doi:10.1016/j.csbj.2022.06.053)
Supplement: Supplementary data 2 [file mmc2.pdf]

Pseudocode of the deconvolution algorithm:

```
Input mitotype Q of putative mixture.
Choose putative number k of components.
Condense the haplogroup motifs to the range of Q.
Initialize
   splittingList=empty,
   mincost1=infinity,
   mincost2=infinity.
For all combinations M1,…,Mk of condensed motifs in graded lexicographic order do:
   Build motif table T of M1,…,Mk.
   Load precomputed list L of irredundant extension tables of T.
   For all tables E in L do:
      Construct from table E the components Q1,…,Qk of the splitting Q=Q1&...&Qk.
      Compute the value c of the splitting costs
         by summing the LLRs between Mi and Qi for i=1,...,k
         until finished or c is equal or greater than mincost2+0.5.
      If c is less than mincost2+0.5 do:
         Insert the item (M1,…,Mk,Q1,…,Qk,c) into splittingList in ascending order of costs.
         Update mincost1 and mincost2 from splittingList.
         Delete all items in splittingList with costs not less than mincost2+0.5.
Build haplogroupList by selecting maximal combinations of haplogroups from splittingList.
For each rank output range of observed costs, splittings in ascending order of costs, and
maximal haplogroup combinations in Phylotree order.
```
